# Tension-Free Vaginal Tape, Transobturator Tape, and Own Modification of Transobturator Tape in the Treatment of Female Stress Urinary Incontinence: Comparative Analysis

**DOI:** 10.1155/2014/347856

**Published:** 2014-03-16

**Authors:** Marcin Zyczkowski, Krzysztof Nowakowski, Waclaw Kuczmik, Tomasz Urbanek, Zbiegniew Kaletka, Piotr Bryniarski, Bartosz Muskala, Andrzej Paradysz

**Affiliations:** ^1^Department and Clinic of Urology, Medical University of Silesia, Katowice, Poland; ^2^Department and Clinic of General and Vascular Surgery, Medical University of Silesia, Katowice, Poland

## Abstract

*Introduction*. This study is a comparative evaluation of the TVT, TOT, and our own modification of TOT (mTOT) in the treatment of female stress urinary incontinence from a single center experience. *Material and Methods*. The study was conducted on 527 patients with SUI diagnosed on the basis of urodynamic studies. They were divided into three groups—TVT: *n* = 142, (TOT): *n* = 129, and mTOT: *n* = 256. All of the patients underwent evaluation at 1, 3, and 6 months after surgery. Results were statistically analysed and compared. *Results*. Objective and subjective effectiveness after the surgery were not significantly different in the study groups and ranged from 90.1% to 96.4%. Mean surgery time was 32.3, 28.2, and 26.4 in the TVT, TOT, and mTOT, respectively. Mean hospitalization time was 2.51 days. Mean catheter maintenance time was significantly higher in the TVT than in other groups. In the TVT group total incidence of complications was 13.4%, and it was significantly higher than that in TOT and mTOT (9.3% and 8.6%, resp.). *Conclusions*. TVT, TOT, and mTOT are highly effective and safe methods in the treatment of SUI. There are no differences in the efficacy between the methods with a little higher percentage of complications in the TVT group.

## 1. Introduction

Stress urinary incontinence (SUI) in women is a widespread disease all over the world. It causes many psychosocial problems and generates significant costs to the budget of health in many countries. In 1993 DeLancey as one of the first researchers concluded that its pathophysiology is associated with a defect in bladder neck and urethra due to the laxity of surrounding tissues and the insufficiency of the internal sphincter of urethra [1]. Various factors may affect the development of SUI. The most well known are vaginal births, overweight and obesity, hormonal disorders, and muscle weakness of pelvic diaphragm. First choice of the treatment for SUI is conservative treatment, whose main elements are lifestyle modifications (physical activity, dietary habits, and weight loss), bladder control exercises, and pelvic floor muscle training (PFMT). In the absence of effects in the conservative treatment including medications and physiotherapy, surgical treatment is necessary. In addition to open techniques such as Burch colposuspension, currently the most often used are minimally invasive methods. Their aim is the suspension of the bladder neck and urethra using synthetic materials, the so-called sling. Abnormal positioning of the urethra and the bladder neck implied the possibility of introducing the method of correcting this condition and in 1996 Ulmsten and colleagues have published the report describing the TVT (tension-free vaginal tape) technique in the treatment of SUI [2]. A few years later, TOT (transobturator tape) method was described in which the tape is carried out between the obturator holes [3]. Both methods are now widely accepted methods of surgical treatment of SUI. The transobturator variant, however, has become more popular nowadays due to similar cure rate with relatively less complications.

## 2. Aim of the Study

The aim of the study is to evaluate the comparative results of surgical management of the stress urinary incontinence in women using tension-free vaginal tape (TVT), tension-free transobturator tape (TOT), and own modification of TOT (mTOT). Study is based on over 10 years of experience from a single center.

## 3. Materials and Methods

**Figure 1:**
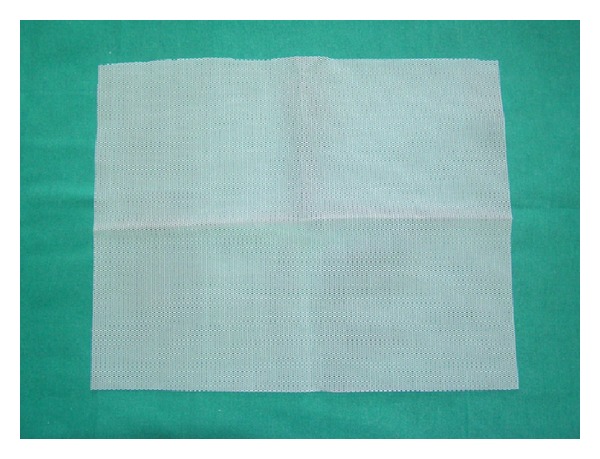
Polypropylene mesh used to prepare TOT tape.

**Figure 2:**
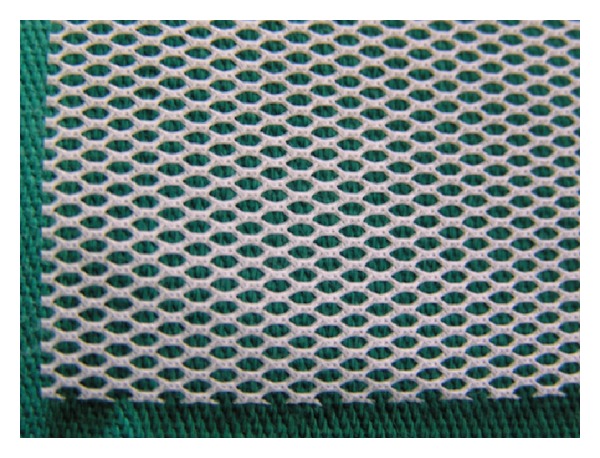
Close-up of polypropylene mesh shown in Figure 1.

**Figure 3:**
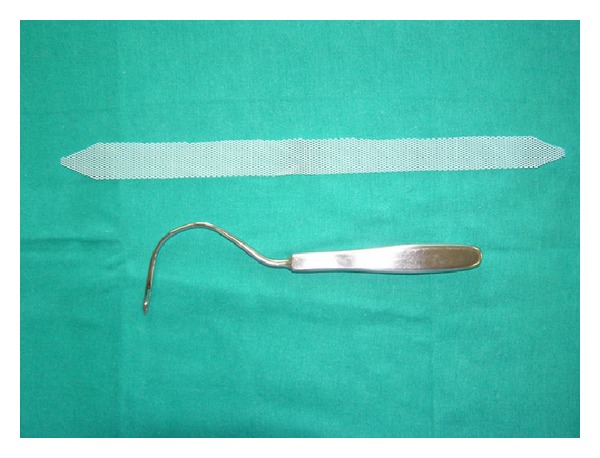
Tape prepared for the mTOT procedure with Stamey needle.

In the years 2001–2012 in Department of Urology in Zabrze, 527 women with SUI were treated using midurethral slings. The age of patients ranged between 45 and 64 years, mean 55.1 years. All patients before treatment were carefully examined and the diagnosis of SUI and qualification for surgical treatment were established on the basis of physical examination, urinalysis and urine culture, abdominal ultrasound, and urodynamic studies in the form of pressure-flow study. 456 patients (86.5%) had pure form of stress urinary incontinence. The remaining 71 (13.5%) presented mixed urinary incontinence (MUI) with urge incontinence component. The treatment option was chosen for the patients according to the routine method used in department at the time when the patients were admitted and treated. Patients were divided into three study groups depending on the type of surgical procedure (Table 1). Group I accounted for 142 patients (26.9%) operated with classical TVT technique. They were treated in the years 2001–2005. Group II consisted of 129 patients (24.5%) who have undergone TOT procedure in the years 2002–2009, using original sets dedicated to this type of treatment. Group III included 256 women (48.6%) who underwent TOT in the years 2004–2012 with our own modification. In that group instead of the original tape dedicated to TOT, self-prepared tape from a polypropylene mesh destinated for abdominal hernias repair was used. It was prepared just before the procedure by the operator from Dallop PP TDM KTM mesh (Figures 1, 2, and 3). All TOT procedures were performed using Stamey needle. The procedures were performed by three different operators with similar experience in the surgical treatment of stress urinary incontinence. Most procedures were carried out under spinal anesthesia (502, 95.3%) and the other in a short general anesthesia due to anaesthesiological indications. Woman with MUI after surgery were subjected to pharmacological treatment with antimuscarinic drugs. Postoperative evaluation was performed after one, three months, and then every 6 months after surgery. The subjective cure rate was evaluated by patients' satisfaction with surgery interpreted as a patient-reported success rate. Patients answered a short questionnaire consisting of three possible answers: (a) I'm very satisfied with treatment results, (b) I'm rather satisfied with treatment results, and (c) I'm not satisfied with treatment results. Answers (a) and (b) were interpreted as patients' related success. Objective cure rate was evaluated on the basis of cough test and one-hour pad test. Completely dry pad after 1 hour of normal day activity was interpreted as a negative result. During the follow-up all of the patients underwent also physical examination, urinalysis, and ultrasound evaluation of postvoiding residual volume. Duration of follow-up was 6–130 months. All data were statistically analysed with Kolmogorov-Smirnov test. For analysis of continuous variables without normal distribution nonparametric *U*-Mann-Whitney test was used. For analysis of categorical variables *χ* ^2^ was used. Statistical examination was conducted with the aid of Statistica Statsoft v 9.0. *P* values <0.05 were considered as statistically significant.

## 4. Results

First evaluation of the cure rates was obtained in 1 month after surgery (Table 2). Objective (cough test and 1-hour pad test) and subjective (patient's satisfaction) cure rates were analysed. There were no statistically significant differences in the efficacy of the surgery between the study groups. Depending on the cure criteria, efficacy ranged from 90.1% to 95.3%, with the highest values in the patients' satisfaction.

Three months after surgery second evaluation was performed (Table 3). Both subjective and objective cure rates were a little higher than 2 months earlier and ranged from 90.8% to 96.4%. No significant differences between the study groups were observed.

Last evaluation of the efficacy was performed 6 months after surgery and it contained the same methods as earlier (Table 4). Subjective and objective cure rates ranged from 91.5% to 96.4% without any differences between the study groups.

Surgery time ranged from 20 to 42 minutes, with means 32.3, 28.2, and 26.4 in the TVT, TOT, and mTOT, respectively. There were no statistical differences between the study groups. Hospitalization time ranged from 2 to 5 days, mean 2.51 days with no significant differences between the groups.

Mean catheter maintenance time in the Group I (TVT) was 1.84 days and it was significantly higher than in TOT and mTOT groups (1.58 and 1.52, resp.). It occurred probably due to higher percentage of bladder injuries in the TVT group.

There were no significant intraoperative and postoperative complications observed in the study groups that required reoperation. Bladder injury, de novo OAB (overactive bladder symptoms), postvoiding residual urine >100 mL, and tape extrusion were analysed as intra- and postoperative complications (Table 5). In the TVT group total incidence of complications was 13.4%, and it was significantly higher than that in TOT and mTOT (9.3% and 8.6%, resp.). The analysis of individual complications showed that the only statistical difference was observed in the frequency of bladder injuries during the surgery and it was highest in the TVT group (4.2%); it occurred only in few cases in TOT and mTOT (0.7% both). De novo OAB occurred in 3.5–4.7% without differences between the groups and was treated pharmacologically after the surgery. Postvoiding residual urine above 100 mL was discovered in the abdominal ultrasound during follow-up in 2.3–4.9%. Tape extrusion was discovered in 5 cases in the whole study group without statistical significance between the groups.

Analysis between TVT and TOT in total (both TOT and mTOT) was also performed in the 6th month after surgery (Table 6). It showed no statistical difference between TVT and TOT in total in both subjective and objective cure rates but it proved higher percentage of complications in the TVT group with statistical significance.

Comparison between TOT and mTOT was also made in the 6th month after the treatment (Table 7). It revealed no differences in cure rates and complications between females operated with classic TOT and TOT with our own modification described above.

## 5. Discussion

World literature reports that TOT has now become a bit more popular, mainly due to the similar efficacy and a slightly lower rate of complications [4, 5]. Published studies show about 80–85% success rate in the efficacy of the TVT method [6, 7]. The most frequently reported complications of this method are bladder perforation, bleeding disorders, and de novo micturition urgency [8]. Published efficacy of the TOT technique is similar, with a slightly lower percentage of complications [9, 10]. Due to the preferences of centers performing such procedures and preferences of individual operators, the number of scientific comparisons of the two methods is low. There are also no standardized uniform methods of follow-up in analysed groups of patients. Depending on study, the level of patients' satisfaction, the results of standardized questionnaires, pad tests, or results of urodynamic studies undergone analysis in the efficacy evaluation. Hence, there are few meta-analyses comparing the efficacy of both methods. In a prospective randomised study comparing the effectiveness of TOT and TVT, based on an analysis of 70 cases, a comparable efficacy of both surgical techniques was achieved, with shorter surgery time and the risk of bladder injury in favor of TOT [11]. Italian researchers, based on a comparative analysis of 148 patients, showed no difference of statistical significance in both—the effectiveness and complication rate between the two groups [12]. In the latest publications from 2013 by Darabi et al., no significant differences in efficacy and safety in both groups were shown, except the bladder catheter maintenance time after surgery [13]. There are a few analyses based on patient-related success rate, same as subjective cure rate in the above article. For example, British analysis from 2012 in prospective randomised controlled trial proved 73% patient-reported success rate for TOT [14]. Large comparative analysis of 1000 cases of SUI treated with TOT and TVT revealed subjective cure rates ranging from 85 to 96% and objective efficacy ranging from 86–91% [15]. Based on 5-year-follow-up evaluation of TOT a study was published in 2013 that showed subjective and objective cure rates at about 90% [16].

The phenomenon of de novo OAB symptoms is a largely debated postoperative complication of midurethral slings. Some studies reported de novo urgency symptoms in 4–33% of operated patients [17, 18]. Confounding role can also play spontaneous development of age-related OAB in a certain percentage of women. In our study de novo OAB was observed in less than 5% of cases and reduced in time during pharmacological treatment.

There have also been some assessments of the results in the treatment of patients with mixed urinary incontinence using midurethral slings. Korean authors concluded in 2003 that treatment with TVT and TOT reduced the percentage of daily incontinence from urgency in these patients, with the higher efficacy in the TVT group [19]. This confirmed the Finnish study from 2013, in which 70% of patients with symptoms of detrusor overactivity declared improvement after the surgery; there was no significant difference between the two operating techniques [20]. A meta-analysis of American scientists in 2007, based on 492 cases, showed no significant differences in the efficacy between the two methods. The conclusion was made that a small number of reports are not clear in showing whether any of these methods are effective in the treatment of urinary incontinence with mixed etiology [4].

The main economical differences between all those three methods were the cost of materials used in the procedure. In mTOT we used a tape made from original mesh used in hernias repair. One tape for mTOT made from it costs around 10 EUR. Original TOT tape in Poland costs about 170 EUR.

## 6. Conclusions

- All sling procedures are effective in the treatment of stress urinary incontinence and in 6th month after surgery achieved cure rates range from 91.5% to 96.4% in subjective and objective parameters of efficacy.
- There are no differences in the efficacy of the treatment of SUI between TVT, TOT, and self-modification of TOT.
- There is a little higher risk of bladder injury during the TVT procedure than in the TOT and mTOT.
- Self-modification of TOT, which consists of self-prepared polypropylene tape instead of original tape, is as effective and safe as original TOT with a lower cost of the procedure.
- Sling procedures in the treatment of SUI are safe and do not cause serious complications.

## Figures and Tables

**Table 1:** Comparison of the study groups.

	Group I (TVT)	Group II (TOT)	Group III (mTOT)	*P* value
Number of patients	142 (26,9%)	129 (24,5%)	256 (48,6%)	
Age	55,2 ± 8,3	54,7 ± 7,8	55,9 ± 8,6	0,115
Complications	19 (13,4%)	12 (9,3%)	22 (8,6%)	0,04
Operation time (min)	32,3	28,2	26,4	0,33
Hospitalization time	2,54	2,48	2,50	0,76
Catheter maintenance	1,84	1,58	1,52	0,04

**Table 2:** Cure rates in 1 month after surgery.

	Group I (TVT)	Group II (TOT)	Group III (mTOT)	*P* value
Cough test negative	128 (90,1%)	118 (91,5%)	236 (92,2%)	0,09
Pad test negative	133 (93,7%)	122 (94,6%)	241 (94,1%)	0,88
Satisfied with surgery	135 (95,1%)	123 (95,3%)	244 (95,3%)	0,91

**Table 3:** Cure rates in 3 months after surgery.

	Group I (TVT)	Group II (TOT)	Group III (mTOT)	*P* value
Cough test negative	129 (90,8%)	120 (93,0%)	238 (92,9%)	0,07
Pad test negative	133 (93,7%)	121 (93,8%)	241 (94,1%)	0,95
Satisfied with surgery	137 (96,4%)	123 (95,3%)	246 (96,1%)	0,88

**Table 4:** Cure rates in 6 months after surgery.

	Group I (TVT)	Group II (TOT)	Group III (mTOT)	*P* value
Cough test negative	130 (91,5%)	120 (93,0%)	239 (93,3%)	0,08
Pad test negative	134 (94,4%)	120 (93,0%)	243 (94,9%)	0,12
Satisfied with surgery	137 (96,4%)	124 (96,1%)	247 (96,4%)	0,98

**Table 5:** Complications.

	Group I (TVT)	Group II (TOT)	Group III (mTOT)	*P* value
Bladder injury	6 (4,2%)	1 (0,7%)	2 (0,7%)	0,01
De novo OAB	5 (3,5%)	6 (4,7%)	11 (4,3%)	0,15
Postvoid residual	7 (4,9%)	3 (2,3%)	7 (2,7%)	0,09
Tape extrusion	1 (0,7%)	2 (1,6%)	2 (0,7%)	0,22

**Table 6:** Comparison between TVT and overall TOT in 6 months after surgery.

	TVT (*n* = 142)	Overall TOT (*n* = 385)	*P* value
Cough test negative	130 (91,5%)	359 (93,2%)	0,07
Pad test negative	134 (94,4%)	363 (94,3%)	0,98
Satisfied with surgery	137 (96,4%)	371 (96,4%)	0,99
Complications	19 (13,4%)	34 (8,8%)	0,01

**Table 7:** Comparison between TOT and TOT with our own modification (mTOT).

	TOT (*n* = 129)	mTOT (*n* = 256)	*P* value
Cough test negative	120 (93,0%)	239 (93,3%)	0,09
Pad test negative	120 (93,0%)	243 (94,9%)	0,88
Satisfied with surgery	124 (96,1%)	247 (96,4%)	0,08
Complications	12 (9,3%)	22 (8,6%)	0,07
